# Episomes and Transposases—Utilities to Maintain Transgene Expression from Nonviral Vectors

**DOI:** 10.3390/genes13101872

**Published:** 2022-10-16

**Authors:** Florian Kreppel, Claudia Hagedorn

**Affiliations:** Chair for Biochemistry and Molecular Medicine, Center for Biomedical Education and Research, School of Life Sciences (ZBAF), Faculty of Health, Witten/Herdecke University, Stockumer Strasse 10, 58453 Witten, Germany

**Keywords:** nonviral vectors, episomes, S/MAR, EBV, transposons, sleeping beauty, gene therapy

## Abstract

The efficient delivery and stable transgene expression are critical for applications in gene therapy. While carefully selected and engineered viral vectors allowed for remarkable clinical successes, they still bear significant safety risks. Thus, nonviral vectors are a sound alternative and avoid genotoxicity and adverse immunological reactions. Nonviral vector systems have been extensively studied and refined during the last decades. Emerging knowledge of the epigenetic regulation of replication and spatial chromatin organisation, as well as new technologies, such as Crispr/Cas, were employed to enhance the performance of different nonviral vector systems. Thus, nonviral vectors are in focus and hold some promising perspectives for future applications in gene therapy. This review addresses three prominent nonviral vector systems: the Sleeping Beauty transposase, S/MAR-based episomes, and viral plasmid replicon-based EBV vectors. Exemplarily, we review different utilities, modifications, and new concepts that were pursued to overcome limitations regarding stable transgene expression and mitotic stability. New insights into the nuclear localisation of nonviral vector molecules and the potential consequences thereof are highlighted. Finally, we discuss the remaining limitations and provide an outlook on possible future developments in nonviral vector technology.

## 1. Introduction

**Figure 1:**
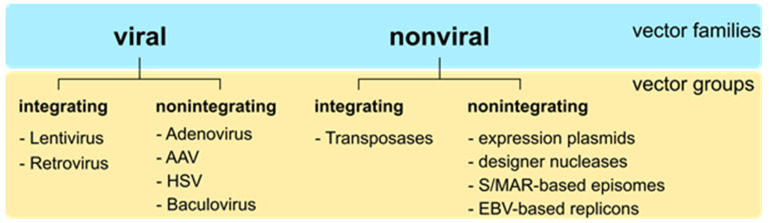
Vector families and vector groups for gene therapy. Vector systems can be classified into families of viral and non-viral vectors. Viral vectors deliver nucleic acids to a variety of cells via transduction. Nonviral vectors are usually delivered using different transfection methods, such as lipofection or electroporation. Both viral and nonviral vectors can be grouped into integrating and nonintegrating vectors. Examples for each vector group are given without any claim of completeness. AAV, Adeno-associated Virus; HSV, Herpes Simplex Virus; S/MAR, scaffold/matrix attachment region; EBV, Eppstein-Barr virus.

Gene therapy is a treatment based on the delivery of genetic material into one person’s cells to prevent or cure diseases, including cancer, genetic diseases, or infectious diseases. As such, gene therapy products are used to either (I) replace a dysfunctional gene, (II) inactivate a disease-causing gene, or (III) introduce a new or modified gene. The number of gene therapy clinical trials increased 8-fold within the last decade (https://www.clinicaltrials.gov/ct2/home, accessed on 22 June 2022). The key step in gene therapy is the safe and efficient delivery of the genetic material to the target tissue/cells. The vehicles used for gene delivery, called vectors, can be either of viral or nonviral origin. Both viral and nonviral vectors can be classified into two groups according to their ability to either integrate into the host genome (e.g., lentiviral vectors [1], sleeping beauty transposon system [2]) or persist as extrachromosomal episomes (e.g., adenoviral vectors [3], S/MAR-based vectors [4], Figure 1).

Viral vectors are, to date, the most efficient delivery systems relying on the natural ability of viruses to deliver their genetic material to recipient cells. Furthermore, vector capsids can be modified to direct vectors to specific target cells or tissues (reviewed in [5]). As such, the majority of ongoing clinical trials are based on viral vectors (www.genetherapynet.com; accessed on 22 June 2022). Nonintegrating viral vector systems, such as adenoviral and adeno-associated virus (AAV) vectors, lack an intrinsic ability to integrate into the host’s genome. Instead, vector genomes remain episomally, thus reducing the risk of insertional mutagenesis. However, these genomes are gradually lost in proliferating cells, as these vectors are usually replication-deficient and, therefore, suitable for disease treatment in post-mitotic tissues [6,7]. Still, gene therapy treatment with these vectors has led to severe adverse effects in clinical studies due to high vector doses of AAV (>10^14^ vg/kg) [8] or the high immunogenicity of adenoviral vectors [9]. The seeming disadvantage of being highly immunogenic is increasingly employed in developing adenoviral vectors as vaccines [10] and oncolytic vectors [11]. A recent study determined integration frequencies of AAV vectors in hepatocytes and detected chromosomal integrations at surprisingly high frequencies (1–3%) in vivo and in vitro. Notably, most of the inserted AAV sequences were rearranged and accompanied by deletions of genomic sequences at the integration site [12]. These results indicate that integration frequencies and, thus, the genotoxicity of AAV and possibly other viral nonintegrating vectors have been underestimated thus far.

Integrating viral vectors, such as retroviral vectors, are equipped for chromosomal integration and are thus mitotically stable in dividing cells resulting in prolonged expression of the therapeutic genes. However, vector insertions occur preferably in actively transcribed genes and have led in the past to genotoxic side effects resulting in the development of leukaemia [13,14,15]. As a consequence, self-inactivating (SIN) lentiviral vectors have been developed that show decreased genotoxicity [16,17,18].

**Figure 2:**
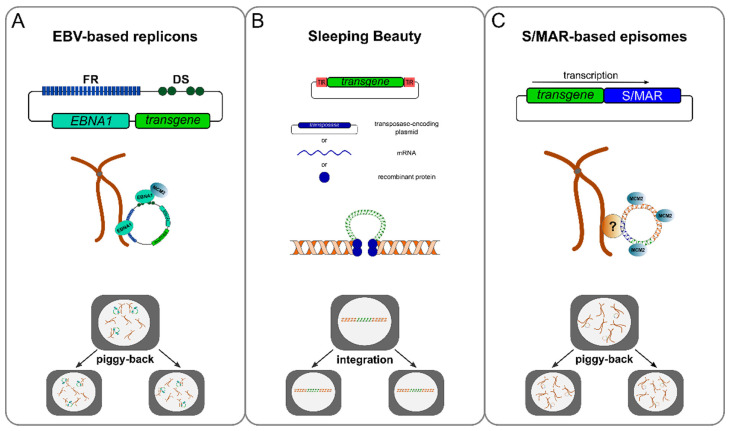
Mechanisms of DNA replication and nuclear retention of different nonviral vector systems. (**A**) EBV-based replicons replicate EBNA1 and MCM2-7 dependent. The binding of EBNA1 to the DS element recruits MCM2-7, thus mediating DNA replication, while binding of EBNA1 to both the FR element and metaphase chromosomes provides a “piggy-back” mechanism for nuclear retention and segregation. (**B**) SB transposases are delivered to the target cells either directly as recombinant protein or as a transposes-encoding plasmid, or mRNA, along with transposon-carrying plasmids (transgene flanked by TIR sites). Once both components (transposon and transposase) are available in the cell, the SB transposase binds to the TIR, excises the transposon and mediates its integration in the cellular genome. Subsequently, the inserted transposon is replicated and segregated during the cell cycle. (**C**) Episomal maintenance of S/MAR-based vectors relies on the presence of active transcription running into the S/MAR sequence. Replication of S/MAR-based episomes is initiated during the early S-phase at various sites of the vector, depending on the host’s cell replication machinery. Segregation and nuclear retention rely on the interaction of the episomes with metaphase chromosomes in a “piggy-back” manner, although the proteins involved are still unknown. EBV, Eppstein-Barr virus; FR, family of repeats element; DS, dyad symmetry element; TIR, terminal inverted repeats; EBNA1, EBV nuclear antigen 1; MCM2, minichromosome maintenance complex component 2; S/MAR, scaffold/matrix attachment region.

So-called viral plasmid replicon-based vectors represent an intermediate between viral and nonviral vectors. Viral plasmid replicon vectors are typically delivered as naked DNA but possess the ability to replicate in the target cell’s nucleus. In contrast to conventional expression plasmids, viral replicon-based vectors exhibit highly efficient strategies to replicate within the nucleus facilitating stable maintenance and prolonged transgene expression in proliferating cells. Of this vector group, Epstein-Barr virus (EBV) derived plasmid replicons have been extensively studied (Table 1). EBV coordinates replication from different origins of replication in lytic and latent infection, respectively. In lytic infections, binding of the viral origin binding protein (OBP) to the lytic origin of replication (ori-Lyt) initiates a rolling circle amplification of the EBV genome. In latent infections, the EBV genome is maintained in infected cells as an episome with approximately 2–20 copies per cell. Here, viral genome replication relies on the binding of the EBV nuclear antigen 1 (EBNA1) to the oriP [19,20,21]. The oriP is a bipartite DNA sequence consisting of a family of repeats (FR) element, harbouring 20 high-affinity binding sites for EBNA1, and a dyad symmetry (DS) element, harbouring four low-affinity binding sites for EBNA1 [22]. The replication of EBV replicons is integrated into the host’s cell cycle by the DS element, whereas the FR element facilitates stable segregation (Figure 2A). Plasmids carrying the DS element and lacking the FR replicate in an EBNA1-dependent fashion but are not stably maintained [23,24]. Smaller versions of the EBV genome lacking the lytic genes have been constructed for gene therapy approaches. However, these mini-EBVs can still transform human B-cells due to the expression of latent genes [25,26] and the EBNA1 itself [27]. Since successful replication and maintenance of EBV episomes is always dependent on oriP/EBNA1, the development of such oriP/EBNA1-only plasmids was a promising attempt at a safe EBV-derived gene vector design [28]. However, the above-mentioned safety concerns, along with observations that EBNA1 might mimic the functions of the cellular high mobility group protein HMG1a [29,30,31], led subsequently to the substitution of viral components of the oriP/EBNA1 system with cellular elements. In principle, these substitutions can be introduced in *cis* and in *trans* and will be discussed in the paragraph *Mitotic Stability*.

Nonviral vectors are considered advantageous due to their safety profile, simplicity, and large-scale producibility. Hence, 20% of all ongoing clinical trials employ nonviral gene transfer. The family of nonviral vectors can be grouped into integrating and episomal vectors. The sleeping beauty (SB) transposase system represents an extensively studied integrating, nonviral vector system (Table 1). Belonging to the Tc1/*mariner* superfamily of class II transposable elements (TE), SB has been reconstructed from fossil DNA sequences of fish genomes [32]. Tc1/*mariner* TE transposition does not rely on an RNA intermediate but occurs directly through DNA instead [33]. The SB transposon encodes for a single gene, the transposase, which enzymatically catalyses the transposition reaction, flanked by transposon-specific terminal inverted repeats (TIR). Expression and binding of the transposase to the TIR induces DNA double-strand breaks (DSBs) at both ends, thus releasing the transposon. Mediated by DNA-repair mechanisms, the DNA-transposase complex then integrates at suitable target sites [34,35]. For gene therapy applications, this system has been modified such that both components of the SB transposase system, the transposase and TIRs, are delivered *in trans*. The transposase gene was replaced with a sequence of interest, e.g., a therapeutic gene, flanked by the TIRs. The transposase itself is provided on a separate expression plasmid [36], as mRNA [37] or as recombinant protein [38] (Figure 2B). Thus, the SB system facilitates transgene insertion and sustained gene expression in the recipient cell.

However, in their simplest form, nonviral vectors are DNA plasmids, often referred to as nonviral episomes, and are among the three most frequently used vectors in clinical trials (www.genetherapynet.com; accessed on 22 June 2022). Nonetheless, these plasmid-based vector technologies usually suffer from inefficient and untargeted delivery (Table 1). Further, most nonintegrating nonviral vectors are not mitotically stable, resulting in the loss of vector molecules as a consequence of cell division and/or degradation. Additionally, transgene silencing, a phenomenon linked to CpG methylation, impedes stable transgene expression [39,40,41]. Therefore, plasmid-based vectors must be equipped with cis-acting sequences to ensure mitotic stability and, thus, prolonged transgene expression.

The characterisation of mapped mammalian origins of replications revealed that so-called scaffold/matrix attachment regions (S/MAR) were often found in close vicinity to replication origins. The insertion of a S/MAR sequence, derived from the human interferon-β gene cluster, into a conventional expression plasmid resulted in the first nonviral vectors shown to replicate autonomously in a variety of different cell lines, including primary cells [42,43]. For episomal replication and maintenance of S/MAR-based episomes, transcription of the S/MAR element linked to an expression cassette is mandatory (Figure 2C). Deleting promoter and/or transcription units or inserting transcription termination signals between transgene and S/MAR led to integration or episome loss after transfection [44]. Transfected in mammalian cells, these S/MAR-based episomes replicate at low copy numbers (1–10 copies per cell) in a once-per-cell cycle manner [45,46]. They are stably maintained in the absence of selection pressure for basically unlimited time [42]. Since these first observations, S/MAR-based episomes have been extensively studied and optimised for their use in gene therapeutic interventions.

An obvious vector for stable large genomic gene loci could be an artificial minichromosome. With the first construction in 1997 [47], human artificial chromosomes (HACs) offered an alternative approach to address the complex requirements of an ideal vector (Table 1). HACs are mitotically and meiotically stable, nonessential, additional chromosomes. They offer a large cloning capacity allowing the insertion of entire genomic loci harbouring all regulatory elements that enable mimicking of the regular pattern of endogenous gene expression. However, for correct replication and segregation during mitosis, the presence of an origin of replication and a centromere are mandatory. Being crucial for the attachment of chromosomes to the mitotic spindle and their subsequent segregation, the centromere is the most important structural feature of HACs. Normal human centromeres contain large arrays of alphoid satellite DNA (α-DNA) that consists of tandem repeats of a 170 bp monomer arranged in highly ordered repeats [48,49]. While only α-DNA efficiently forms centromeric structures after being delivered to mammalian cells, not every α-DNA can form active centromeres [47,50]. It further appeared that functional centromeres require a minimum size of approx. 100 kb [51], with the exception of α-DNA from chromosome 21, which could vary between 50 and 100 kb [52]. As exogenous minichromosomes, HACs are generated using either a “top-down” (engineered chromosomes) or a “bottom-up” (*de novo* artificial chromosomes) approach. *De novo* HACs are generated in the recipient cells by transfecting a vector equipped with the essential centromeric chromosomal sequences [47] and subsequent recombination [53]. Using this approach, HACs establish as circular chromosomes and thus circumvent the need for telomeric sequences. The delivery of HACs usually relies on polyamines and lipofection, followed by microcell-mediated chromosome transfer (MMCT). However, these techniques share a limited efficiency. As such, different delivery strategies were explored, utilising either the fusion machinery of viruses [54] or virus-based amplicons [55]. While the development and improvement of HACs experienced significant progress within the last decade [56,57,58,59], their design and construction require particular considerations and utilities that differ from those needed for nonviral vectors. As these have been comprehensively reviewed elsewhere [60,61,62], we will focus in this review on the three nonviral vector systems EBV-based replicons, Sleeping Beauty, and S/MAR-based episomes.

As outlined above, long-term nuclear maintenance and stable transgene expression in therapeutically relevant cell types and tissues is a major challenge in nonviral vector development. Accordingly, significant efforts have been made to address these hurdles. In this review, we will discuss recent progress in nonviral vector development regarding (I) transgene expression, (II) mitotic stability, and (III) nuclear localization.

## 2. Transgene Expression

The stable and controlled expression of a therapeutic gene independent of the potency of the target cell to proliferate is critical for lasting therapeutic success, particularly in gene replacement approaches. Thus, one major challenge is to achieve stable long-term transgene expression. The choice of the transgene promoter type has the most fundamental impact on transgene expression levels. Most promoters used in viral and non-viral vectors are variants of viral or cellular constitutive promoters and exhibit distinct advantages. The cytomegalovirus (CMV) early enhancer/promoter facilitates a high level of transgene expression in various cell types [63,64,65]. Thus, this promoter is most commonly used in nonviral vectors [42,43,44,66]. Linking the CMV promoter with an intron, placed between promoter and transgene, was shown to additionally increase transgene expression levels in S/MAR-based episomes [67]. However, the CMV promoter is highly prone to silencing over time, resulting in the loss of transgene expression. Although this effect may vary between different cell types [68,69], other constitutive promoters such as the cytomegalovirus early enhancer/human elongation factor-1α (*CMV/hEF-1α*) and cytomegalovirus early enhancer/chicken β-actin (CAG) promoters have been proven to mediate stable transgene expression for long periods of time. Replacing the CMV promoter with a CMV/hEF-1α promoter significantly increased and prolonged transgene expression in vitro and in vivo [70]. In contrast to the CMV promoter, the CAG promoter is less prone to cytosine methylation [71]. It has been successfully used for the generation of induced pluripotent stem cells (iPSCs) [72] and the genetic engineering of stem cells [73].

High-level transgene expression may have toxic effects depending on the nature of the transgene and the recipient cell or tissues [74]. Here, low-yield endogenous promoters such as the human 3-phosphoglycerate kinase (PGK) or hEF-1α promoters have been successfully used to stably express transgenes in patient-derived primary cells [75] and stem cells [76]. However, some gene therapy applications require a tissue-restricted expression of the transgene. While viral vectors naturally have a limited tropism or can be modified to target specific tissues [5], nonviral vectors are usually delivered as naked DNA molecules. Here, a restricted transgene expression can be achieved by using tissue-specific promoters. For example, replacing the constitutive CMV promoter with either the liver-specific human α anti-trypsin (hAAT) promoter, the muscle-specific smooth muscle 22 (SM22) promoter, or the tumour-specific α-fetoprotein (AFP) promoter led to tissue-specific transgene expression in liver, muscle cells, and AFP-positive carcinoma cells, respectively [77,78].

Compared to strong viral promoters such as CMV, endogenous or tissue-specific promoters are often characterised by lower expression levels. Thus, combining a weak or tissue-specific promoter with enhancer elements mediated strong transgene expression and sustained tissue-specificity [78]. However, due to safety concerns, considerable efforts have been made to substitute viral enhancer sequences with cellular elements that avoid the spread of heterochromatin and hypermethylation. Several different of such protective elements, such as the 5′HS chicken β-globin (cHS4) insulator [79,80,81,82], the D4Z4 insulator [82], matrix attachment regions (MAR) [83,84], and Ubiquitous Chromatin Opening Elements (UCOE) [81,82], have already been exploited for transgene protection in both, viral and non-viral vector systems. The UCOE elements have been identified within the *TBP-PSMB1* and *HNRPA2B1-CBX3* gene loci. These loci’s dual divergently transcribed promoter regions could confer stable gene expression even from within a centromeric heterochromatic environment [85]. The UCOE derived from the *HNRPA2B1-CBX3* locus (A2UCOE) significantly improved transgene expression concerning expression levels and duration [86]. In viral vectors, UCOEs have been shown to mediate both enrichment of permissive histone marks (e.g., H3K4me3) and hypomethylation [87,88]. Inserted upstream of the CMV promoter in the context of a S/MAR-based nonviral episome, the 4 kb-A2UCOE element led to increased and homogeneous transgene expression within a cell population [81]. Likewise, in the context of SB transposon vectors, introducing a 1.5 kb-UCOE element immediately upstream of a CMV promoter led to reduced transgene silencing. Moreover, a core fragment within the 1.5 kb-UCOE was identified using bioinformatic approaches. This core fragment, only 0.86 kb in size, offered effective protection from transcriptional silencing [82].

Other cis-acting elements, such as insulators, have also been successfully used to shield transgene cassettes from silencing. The DNAseI hypersensitive site of the chicken β-globin locus control region (cHS4) exhibits classical insulator properties. The cHS4 insulator has been shown to block enhancer activity and functions as a barrier. The cHS4 recruits several DNA binding proteins, of which the CCCTC-binding factor (CTCF) has been linked to its enhancer-blocking function [89]. Other proteins, such as the Poly(ADP-ribose) Polymerase-1 (PARP-1) and the upstream stimulatory factors 1 and 2 (USF1 and 2), are presumably involved in the insulator’s barrier activity [90,91]. Besides its enhancer-blocking and barrier activities, evidence suggests that the cHS4 insulator is also involved in nuclear organisation and can anchor active domains to specific subnuclear compartments [92,93]. In SB transposon vector context, cHS4 insulator sequences flanking the transgene expression cassette increased expression level and reduced interclonal variation in CHO cells [82] and murine embryonal carcinoma cells [79].

In summary, these results clearly show that a stable and prolonged transgene expression significantly depends on the choice of promoter and subsidiary cis-acting elements. Here, the promoter and, if necessary, a respective cis-acting element should be carefully chosen depending on the desired application, targeted tissue and desired transgene expression level.

## 3. Mitotic Stability

### 3.1. Viral Elements That Confer Mitotic Stability

Stable maintenance of nonintegrating vectors requires both replication and segregation of the vector genomes synchronous to the cell cycle. While for integrating nonviral vector systems, such as SB transposons, stable vector genome maintenance is facilitated via integration, episomal vectors rely on certain genomic elements facilitating replication and segregation. As outlined above, the EBV-derived oriP/EBNA1 vector system relies on viral elements that facilitate vector genome replication (DS element) and segregation (FR element). However, safety concerns led to attempts to substitute some of these viral components with cellular elements, both *in cis* and *in trans*. It has been shown that replication of the EBV genome can be initiated at sites other than the DS-element [94]. Consequently, it is dispensable for stable maintenance of the whole EBV genome [95]. For Mini-EBV genomes, Ott et al. demonstrated that replication is initiated primarily in the close vicinity of the DS-element when its position has been altered. Moreover, when completely deleted, replication starts throughout the mini-EBV genome at weaker replication origins [96]. However, for oriP-only plasmids, the DS element is mandatory for stable episome maintenance and thus needs to be replaced. Cellular sequences with replication competence, termed origins of replications, are thus favourable for substitution. Yet, analyses of mapped mammalian origins of replications have not revealed any consensus sequences. Instead, AT-rich sequences, CpG-islands, bent DNA and the presence of S/MAR sequences are common structural features within origins [97]. Using a so-called origin-trapping assay, Gerhardt et al. isolated potential origins bound by Orc2 by ChIP-assay. Orc2 is a protein of the replication machinery involved in assembling the pre-replication complex (preRC). Subsequently, these sequences were cloned in oriP plasmids lacking the DS element and analysed for their replication competence. They demonstrated that the DS element could be replaced with cellular sequences, and, remarkably, these sequences were all located at a distance of 7 to 10 kb from matrix attachment regions. Moreover, some identified sequences could facilitate replication independent of EBNA1 [98].

Structural analyses of the EBNA1 protein identified the N-terminus being critical for chromosome binding while the C-terminus enables the accumulation of replicated oriP-plasmids in the nucleus. High mobility group (HMG) proteins contain AT-hook motifs. Especially one member, HMGA1, binds highly specific to the minor groove of AT stretches, thus inducing conformational changes [99]. Such AT-hooks have also been identified within the N-terminus of EBNA1 [30]. Replacing the EBNA1 N-terminus with cellular proteins revealed that amino acids 1–90 of high mobility group-I protein and histone 1 could substitute the EBNA1 N-terminus and mediate association with mitotic chromosomes, nuclear retention, and long-term maintenance [29]. In a subsequent study, an HMGA1:EBNA1 fusion protein was employed to analyse the effects of HMGA1 on episome replication. This fusion protein mediated replication of an oriP-plasmid in an Orc-dependent manner. In fact, HMGA1:EBNA1 binds specifically to Orc, thus generating a functional origin of replication. Fusing HMGA1 to tetracycline repressor (TetR) could facilitate plasmid replication when the DS-element was replaced by four tet-operator (tetO) sites [31]. Using a similar approach, Pich et al. constructed a conditionally replicating oriP-vector. The 20 EBNA1 binding sites of FR were replaced with 20 TetR binding sites. Accordingly, the EBNA1 N-terminus was fused with TetR. When doxycycline is added to the system, the TetR domains undergo conformational changes and can no longer bind to the tetO sites. In the absence of doxycycline, binding of EBNA1:TetR to the modified oriP facilitated DNA replication and episome maintenance [100]. In a combinatorial approach [31], the FR and DS elements were replaced with tetO sites. This artificial oriP-vector was maintained episomally and became almost undetectable when doxycycline was added to the cells [100].

### 3.2. Chromosomal Elements That Confer Mitotic Stability

S/MAR-based vectors were the first vectors lacking viral elements. They were first introduced in the late 1990s and rely on a S/MAR-sequence, derived from the β-interferon (IFNβ) gene locus, linked to a transgene expression cassette [42]. These episomes establish with low copy numbers (1–10 copies per cell) in the nucleus [46] and are mitotically stable for basically unlimited time [42]. The S/MAR-episomes are associated with the nuclear matrix via the SAF-A protein [101,102]. The replication of S/MAR-based episomes occurs in a once-per-cell cycle manner during the early S-phase, and proteins of the preRC have been shown to assemble at various sites throughout the vector [45]. Since the mitotic stability of S/MAR-based episomes relies on an ongoing transcription into the S/MAR [44], switching off transgene expression would lead to episome loss. Using a doxycycline-inducible promoter (TetOn), Rupprecht et al. demonstrated in a proof-of-concept study that the removal of doxycycline resulted in a gradual loss of vector molecules in vitro and in vivo [103]. Since the efficiency with which S/MAR-based episomes are established in the nucleus is comparably low [46], various attempts focused on identifying cis-acting elements supporting establishment efficiency. While the original INFβ-S/MAR spans 2 kb, Jenke et al. designed a minimal S/MAR module (155 bp) that comprises the core unwinding element of the upstream IFNβ-S/MAR. Inserted as a dimer or tetramer downstream of the transgene, only the tetramer facilitated episomal maintenance, while the dimer integrated into the host’s genome [102]. Another approach is based on the observation that the cHS4 insulator anchors active domains to specific subnuclear compartments [92,93]. Thus, the 1.2 kb cHS4 was inserted downstream and in a reverse orientation of the S/MAR. These S/MAR-cHS4 episomes showed up to 2.5-fold increased establishment efficiencies compared to S/MAR-based episomes without the cHS4. In the episome context, the binding of CTCF was mapped exclusively to the 5′-region of cHS4 and is thought to act as an additional anchor for these episomes [81].

Although the original S/MAR-based episome [42] was shown to establish in primary CD34^+^ cells, only 1% of progeny cells stably maintained the S/MAR-based episome [43]. To overcome this limitation, a bona fide mammalian origin of replication, the β-globin replicator, was employed. Being the replication initiation region (IR) of the β-globin gene locus, this element was inserted upstream of the promoter. The IR-S/MAR vectors were established episomally in CD34^+^ cells with significantly increased frequencies [104]. Additionally equipped with an artificial transcription activator that binds specifically to the ^A^γ-globin promoter, Stavrou et al. demonstrated the efficient induction of γ-globin transcription. This development may contribute to effective gene therapy for treating β-thalassaemia and sickle cell disease [105]. While S/MAR sequences linked to an expression cassette facilitate episomal maintenance of plasmids, they have also been studied for their effect on SB transposition activity. Inserting a S/MAR sequence derived from the human β-globin locus upstream of the transposase expression cassette improved transposition frequency and, thus, long-term transgene expression [83].

Another structural feature tightly linked with DNA replication is the so-called G-quadruplex (G4) structure. These secondary DNA structures are constructed from guanine-quartet-building blocks forming square-planar assemblies of four Hoogsteen-bonded guanine bases [106]. Potential G4-forming sequences (pG4) are not distributed randomly throughout the genome but tend to accumulate in promoter regions and 5′-and 3′-untranslated regions of mRNA [107]. Origin G-rich Repeated Elements (OGRE) are considered pG4s and are present in more than 60% of origins in fly, mouse, and human cells [108,109]. They are located upstream of the replication initiation site, compatible with the position of the preRC [110]. As such, plasmids equipped with an OGRE element could replicate in HEK293 cells that express EBNA1 almost as efficiently as plasmids containing the oriP. Accordingly, the deletion of the OGRE element strongly reduces its replication efficiency [111]. This is particularly interesting since the EBNA1-mediated recruitment of Orc to the DS element has been linked to an RNA-dependent mechanism [112]. The EBNA1 linking regions 1 and 2 (LR1 and 2) have been shown to bind G-rich RNA that is predicted to form G4 structures. G4-interacting drugs, such as BRACO-19, consequently disrupted the interaction between EBNA1 and Orc, inhibited EBNA1-mediated replication, and interfered with the ability of EBNA1 to tether to metaphase chromosomes [113]. The introduction of a pG4 oligonucleotide into S/MAR-based vectors immediately upstream of the promoter led to stable maintenance even when the S/MAR element was deleted (Hagedorn and Lipps, unpublished results). These observations suggest that episomal replication and maintenance might also be mediated through G4 structures.

### 3.3. Modifications to Minimize Effects of Innate Immune Responses

In eukaryotes, CpG dinucleotides appear with a frequency below their statistical expectations and are often methylated to *5-methyl-cytosine* (^m^CpG). However, some genomic regions, termed CpG islands, contain atypical high frequencies of CpG dinucleotides that generally lack methylation [41]. In bacterial genomes, CpG dinucleotides are represented with a frequency reflecting their statistical expectation and are usually unmethylated [114]. Thus, mechanisms of the human innate immune response to differentiate between bacterial and own DNA rely on CpG dinucleotide occurrence and methylation. The toll-like receptor 9 (TLR9) interacts with unmethylated CpG dinucleotides in DNA molecules and subsequently activates inflammatory downstream signalling involving MyD88. This increases NFkB and AP-1 expression, resulting in the production of inflammatory cytokines [115,116] and might lead to vector loss and/or transgene silencing. Remarkably, one single CpG dinucleotide in a DNA vector triggered inflammatory responses in vivo [117]. These understandings led to the development of CpG-depleted DNA plasmids, thus minimising silencing phenomena and undesired stimulation of the innate immune system [117,118]. Consistently, S/MAR-based episomes with reduced CpG content in the backbone have been constructed. Further, the CpG-depleted vector backbone was combined with a hCMVe/EF1a promoter that is less prone to silencing [117]. This novel class of S/MAR-based episomes, referred to as pEPito, showed enhanced transgene expression and increased colony formation efficiencies, indicating improved mitotic stability [70]. Accordingly, episomal vectors lacking any residual bacterial sequences, so-called minicircles, should be able to avoid innate immune responses [119]. The first S/MAR-based minicircles were generated using the Flp-recombinase technique producing two circular units: a miniplasmid containing the bacterial vector elements and a promoter-transgene-S/MAR minicircle. Like the parental S/MAR-based episomes, these minicircles are mitotically stable and showed enhanced transgene expression and establishment efficiencies in vitro and in vivo [77,120].

Nevertheless, the production of minicircles is a time-consuming process that involves intramolecular recombination. Antibiotic-free (AF) minimally sized vectors have been developed as an alternative. The AF selectable vectors either rely on RNA-interference [121] or amber-suppressor tRNAs [122]. Equipped with the IFNβ-S/MAR sequence, these AF S/MAR vectors produced more robust transgene expression and showed increased establishment efficiencies in dividing cells [123]. Combined with the liver-specific HCRHPi promoter that contains the apolipoprotein hepatic control region (ApoE-HCR), increased and stable transgene expression restricted to liver cells was shown [124]. Yet, aside from a proof-of-concept, AF S/MAR vectors have been reported for their application in the genetic modification of primary pancreatic cancer cells. Besides the sustained supplementation of a tumour suppressor, in terms of genotoxicity, only minimal impact on the target cell genome was detected [123].

Similar modifications have also been applied to SB vectors. The success of SB-mediated gene therapy strongly depends on the efficient delivery of the plasmid DNAs and subsequent transgene integration rates. Therefore, applying the minicircle technique to the SB transposase vector system is cogent. Converting the SB transposon system into a minicircle combined with the hyperactive SB100x enhanced integration properties in human haematopoietic stem cells (HPSCs) [36]. Further, delivery of both SB100x transposase and transgene using an AF minimally sized plasmid significantly increased transgene integration efficiencies [125]. This is particularly interesting since the number of integrated transposon copies correlates linearly with transgene expression [126].

Recently, the group of Harbottle fundamentally modified S/MAR-based episomes, developing a new generation of S/MAR vectors termed nano-S/MARs (nS/MAR) [73,75]. The prokaryotic vector backbone was depleted for CpG sites, and inserting an RNA OUT element [121,123] enabled antibiotic-free selection. Further, the IFNβ-S/MAR was replaced with a more compact version isolated from the apolipoprotein B (ApoB) gene cluster. Stretches of ATTA-ATTTA are the predominant motif of matrix attachment sites and are recognised by homeodomain proteins [127]. The ApoB S/MAR comprises a stretch of 555 bp, almost entirely composed of these motifs. Since the mitotic stability of S/MAR-based vectors relies on transcription running into the S/MAR sequence, a long mRNA containing the S/MAR sequence is transcribed [46]. However, AU-rich elements within the untranslated regions of mRNAs serve as signals for rapid degradation mediated by the human antigen R [128]. This would impair transgene expression while the vectors are still episomally maintained. In a very sophisticated approach, Bozza et al. addressed this problem by introducing splice sites flanking the S/MAR [75]. The nS/MAR were maintained episomally, displayed highly stable transgene expression, and were established most efficiently compared to previous vector generations. While this approach was successful for intron-less transgenes, it should be noted that the use of intron-containing transgenes (e.g., whole genomic loci) might yield unfavourable splicing variants and, thus, might not be feasible. The exact mechanism underlying the transcription-coupled mitotic stability of S/MAR vectors is still not fully understood. However, these results indicate that an RNA-dependent mechanism might be involved, as described for EBNA1 (90,91).

Moreover, nS/MAR vectors were employed to generate CAR-T cells. In a side-by-side comparison with a lentiviral vector, nS/MAR vectors had only a minimal impact on human T-cell gene expression. They were shown to be suitable for large-scale GMP-compatible production of CAR-T cells. Finally, nS/MAR engineered T-Cells mediated tumour killing in vivo with an efficacy comparable to CAR-T cells modified with a lentiviral vector [75]. In the same group, nS/MAR vectors were exploited for the genetic modification of pluripotent stem cells (PSCs). Remarkably, the nS/MAR vectors did not impair the pluripotency of the modified PSCs and persisted during reprogramming and differentiation, both in vitro and in vivo [73].

Interestingly, all nonintegrating, nonviral vectors and minicircles are maintained with a similar copy number in the nucleus [46,75,81,104], suggesting stringent copy number control in the recipient cells. Thus, epigenetic features, such as chromatin structure and nuclear localisation, seem to be essential in regulating the establishment of nonviral episomes.

## 4. Nuclear Localisation

Within a eukaryotic nucleus, the genome is compartmentalised. Chromosomal regions with similar functional properties (e.g., heterochromatin and euchromatin) often cluster together, forming distinct compartments referred to as the A- and B-compartments [129]. Further, during interphase, chromosomes are partitioned into topologically associated domains (TADs). These domains are characterised by intensive internal chromatin interactions and fewer contacts to neighbouring regions [130]. Today it is widely accepted that the spatial organisation of the genome and transcriptional activity are tightly liked [131,132].

The stable establishment of S/MAR-based vectors in the nucleus is a rare event. Only 1–5% of all initially transfected cells stably establish episomal vectors as autonomous replicons [81,102]. The episomal establishment is considered a stochastic process that presumably depends on the nuclear compartment a vector molecule reaches after transfection. However, the underlying mechanisms are still poorly understood. The nuclear localisation of S/MAR-based episomes has been extensively studied using fluorescence in situ hybridisation and immunofluorescence. Established episomes co-localised in the nucleus with chromosomal domains that harboured active histone marks, such as acetylated H3K9 and K14, associated with active promoters and enhancers [46,133]. Further, episomes were found in close proximity to nuclear speckles and early replication foci. Neither an association with repressive chromatin marks nor specific chromosomes was detected [46]. Using chromatin-immunoprecipitation (ChIP) assay, Rupprecht et al. monitored the association of S/MAR-based vectors with certain histone marks throughout the cell cycle. In S-phase, the chromatin structure of S/MAR episomes was marked by H3K4me1 and H3K4me3. Both histone modifications are associated with transcriptionally competent chromatin [134,135] and were predominantly enriched in the 3′-region of the S/MAR. Interestingly, during mitoses, most histone modifications analysed in this study were depleted [136].

As different as EBV-derived and S/MAR-based vectors are regarding the mechanisms facilitating their mitotic stability, they have much in common regarding their nuclear localisation. The establishment process of EBV-derived vectors relies on the efficacy of initiating DNA replication [137]; a prerequisite for efficient replication is the sufficient tethering of oriP-plasmids to chromosomes. However, tethering to specific chromosomes has not been described [138]. Within the nucleus, EBV-genomes were described to co-localise with chromatin domains enriched for active histone modifications (H3K4me3 and H3K9ac) but not with nuclear speckles. Strikingly, enrichment of H3K4me3 histone modifications was detected within the oriP of the EBV genomes [137]. This is particularly interesting as a similar enrichment was detected for the S/MAR sequence [136], being the functional unit of S/MAR-based vectors.

More recently, using circular chromosome conformation capturing (4C), the genomic contact sites of three different S/MAR-based vectors were mapped. As previous results indicated, all S/MAR-based vectors interacted with genomic loci enriched for histone marks of open chromatin (e.g., H3K36me3, H3K27ac, H3K4me2) and markers associated with replication initiation (H3K79me2). Further, S/MAR-based vectors preferentially interacted with promoters and transcription start sites. Accordingly, within the genomic contact sites, repressive chromatin marks and transcription end sites were diminished [67]. Even though the epigenetic signature of the chromosomal contact sites was coherent, S/MAR-based vectors displayed an individual chromosomal contact pattern depending on inserted cis-acting elements. While the parental S/MAR-based vector exhibited few and non-clustered contact sites, those of an episome harbouring the cHS4 insulator downstream of the S/MAR [81] appeared clustered to distinct chromosomal loci. In contrast, genomic contact sites of an intron-carrying S/MAR-based episome were more frequent and evenly scattered throughout the genome [67]. It is conceivable that the distinct pattern of genomic contact sites reflects co-transcribed sequences in specialised transcription factories [139,140,141]. Thus, these results indicate that the genetic composition of an episome influences its nuclear localisation and the contact sites it preferentially associates with. This knowledge might lead in the future to the design of directed nonviral episomes, a perspective that has already been realised for SB vector systems.

High-resolution, genome-wide mapping of SB integration sites revealed a close-to-random insertion profile concerning genes and chromosomes [126,142]. A side-by-side comparison of SB, MLV retrovirus integration sites, piggyBac (PB) transposase, and HIV lentivirus revealed unequal biases across the four systems concerning integration in genes previously linked to genotoxicity. While PB, MLV and HIV were preferentially inserted into expressed genes, SB revealed a close-to-random insertion profile, supporting the relative safety of this vector system among integrating vectors [142]. A similar insertion profile has been detected for a sandwich version of SB (SA) that carries two complete transposon elements in head-to-head orientation, flanking the expression cassettes [143]. Again, the SA vector system displayed a close-to-random insertion profile with a slight overrepresentation of repetitive elements, such as satellites, LINS and small RNA genes [126]. Of note, the relatively safe SB insertion profile is independent of its delivery. The insertion profile remained close-to-random when delivered with adenoviral or lentiviral vector systems [144,145,146,147]. However, even with a close-to-random insertion profile, the interactions between integrated transgenes and genomic insertion loci are hard to predict. The position of the transgene within the genome affects not only transgene expression but also, owing to circumstances, endogenous gene expression [14,148,149,150]. Thus, the possibility of a directed insertion would be most desirable. First attempts employed custom DNA binding domains (DBD) engineered to specifically bind to a genomic DNA sequence of choice. Mainly two strategies were applied: direct fusion of such engineered DNA binding protein (DBPs) to the SB transposase or fusing the DBPs to adaptor proteins that interact with the transposase or transposon [151,152,153]. A recent study combined a catalytically inactive Cas9 protein (dCas9) with the SB100x transposase or an N-terminal fragment (N57) encompassing the DNA-binding and dimerisation functions. By generating guide RNAs (sgRNAs) to target the HPRT gene and AluY, directed SB-mediated insertion could be analysed. While enrichment for dCas9-N57 targeting AluY was relatively weak, enrichment dCas9-SB100x was more pronounced and occurred in the vicinity of sites specified by the sgRNA. However, no insertion at the HPRT locus was detected within 5 kb in either direction. It is speculated that either targeting a single-copy locus is not possible with this system or the number of insertion sites was too low to provide the necessary resolution for mapping [154]. However, despite these promising attempts, targeted insertion approaches still face some hurdles. The direct fusion approach often led to reduced transposase activity, and targeting efficiency, in general, was relatively low compared to untargeted background integration (reviewed in [155]).

As mentioned above, a significant safety concern of integrating vector systems is the possibility of genotoxicity. However, the close vicinity of episomal vectors to actively transcribed chromatin regions raises the question of whether the pure presence of a transcriptionally active yet episomal unit may impact host gene expression. This concern is supported by the observations that transcription is a major force in shaping the spatial organisation of the human genome (reviewed in [131,132]). Consequently, careful transcriptome analyses of cells modified with S/MAR-based vectors were performed. In established cell lines and primary cells, only minimal impact on the host cell’s endogenous gene expression was monitored [67,73,75]. This minimal impact was further reduced when next-generation nS/MAR vectors were used [73], which are depleted for CpG sites and dispensable prokaryotic sequences. Gene ontology analyses did not reveal specific genes deregulated by S/MAR-based vectors, suggesting that the gene groups being up- or down-regulated are cell-type- or transgene-specific [67,73,75]. However, as an analogue to the genotoxicity of integrating vectors, it is yet unknown whether episomes tethered to chromosomes impact the genomic stability of the cell. As such, monitoring the incidence of chromosomal rearrangements and aneuploidy in cells stably maintaining episomal vectors compared to unmodified cells would give first hints on the existence of episomal mediated “chromotoxicity” and should be addressed in future studies.

## 5. Nucleic Acid Delivery

One common theme for all the diverse systems mentioned above is the roadblock of specific and efficient delivery to target cells. Delivery vectors that are derived from virus capsids and/or incorporate viral functions for, e.g., endosomal escape and nuclear import, typically exhibit reasonable gene transfer efficiencies in vitro but often suffer from immunogenicity, toxicity, unwanted sequestration, and mistargeting in vivo. Nevertheless, several technologies have been developed during the last decades to improve specific targeting and/or shielding of such vectors. This research area is out of the focus of this review, and we would like to (subjectively and exemplarily) redirect the reader to [156,157,158].

One delivery vector, however, deserves explicit mention here since it has been used in presumably hundreds of millions of humans world wide with distinct success: the so-called lipid nanoparticle (LNP). It has been used in slightly different forms as the delivery vehicle for mRNA-based vaccines against COVID-19 [159,160]. Lipid nanoparticles form lipid envelopes that compact and protect nucleic acids for delivery [161]. The use of different lipid components influences the stability and fusion properties of these lipid nanoparticles. Importantly, by using shielding reagents, protein and non-protein targeting ligands, and different bioresponsive chemical bonds that enable environment-specific dynamics, very specific delivery vectors can be tailored [162]. Thus, it appears likely that once the genetic material can be successfully delivered to the nucleus, the systems discussed in this review might be delivered both in vitro and in vivo by optimized lipid nanoparticles.

## 6. Discussion and Outlook

Gene therapy treatments are complex procedures and are still associated with some risks. Given the predominance of delivery efficiency, most approved gene therapy products are based on viral vectors. Although several challenges remain to be solved, viral vectors bear the intrinsic capability of introducing genetic material into cells with high specificity and efficiency. Thus, viral vectors are suitable for genetic modifications in vitro and in vivo. Nonviral vectors and episomes have also been substantially improved within the last decade. However, of the three vector systems discussed in this review, only the SB vector system is currently being tested in several clinical trials (reviewed in [163]), all focusing on T-cell modifications. Despite many advances and significant improvements, the efficient and specific delivery of these vectors is still a major challenge. Usually, nonviral vectors are delivered by different transfection methods, like lipofection and electroporation, that are comparably inefficient and not applicable for targeted in vivo delivery. Thus, the development of hybrid viral vectors combining the specificity and efficiency of the delivery of viral vectors with the relative safe long-term maintenance of nonviral vectors was a huge step forward in the field. In fact, S/MAR-based episomes, EBV-based replicons and SB have been adopted for viral vectors [144,145,146,147,164,165,166,167,168,169,170]. Combining lentiviral delivery with transposase-mediated transgene integration only slightly reduced the transduction efficiency of lentiviral vectors but resulted in safer integration profiles compared with lentiviral-mediated integration [144,145,146]. The adoption of SB for adenoviral or baculoviral delivery enabled in vivo delivery of SB transposases in the murine and canine liver [169] and mouse eye [168]. Again the integration profile was similar to that observed for nonviral SB vectors [147]. Likewise, the quantitative delivery of oriP/EBNA1 episomes was improved when delivered with adenoviral vectors, resulting in stable long-term transgene expression, both in vivo and in vitro [164,165,166]. An adenoviral-S/MAR hybrid vector was developed to synergise the efficient delivery of adenoviral vectors with the episomal maintenance of S/MAR-based episomes. This hybrid vector system was delivered highly efficient and facilitated episomal maintenance and long-term transgene expression both in vitro and in vivo [170]. Equipping an adeno-associated viral genome with a S/MAR sequence facilitated long-term maintenance of the vector-hybrid genome in mitotic cells [167].

Despite these promising approaches, a literature search (August 2022) revealed that the development of hybrid viral vectors peaked between 1999 and 2016, with approximately 86 publications per year. Still, no significant contributions to the field were found thereafter. The reasons for this stagnation remain speculative. Viral hybrid vectors are rather complex compared to their current nonhybrid counterparts. Thus, the process of adopting nonviral systems to viral delivery and the subsequent production of those hybrid vectors can easily become a highly demanding endeavour. Equipping an AAV vector genome with a S/MAR sequence resulted in episomal maintenance in dividing cells. Yet, the initial establishment efficiency of AAV-S/MAR episomes was approximately ten times lower compared to standard S/MAR-based episomes [167], indicating that additional and system-specific modifications are necessary when adopting nonviral vectors for viral delivery. Another factor that might contribute to the stagnation in the development of hybrid vectors is the lack of comprehensive safety profiles. Nonhybrid vectors have been successfully used in clinical trials, and their safety profiles are extensively studied. However, thus far, only little is known regarding the safety profile of hybrid vectors. For example, adopting S/MAR-based episomes for AAV- or Ad-mediated delivery involves the transformation of an initially circular episome to a linear viral genome. S/MAR sequences confer episomal maintenance when inserted downstream of an expression cassette [44]. However, positioned upstream of an expression cassette, these sequences have also been shown to increase chromosomal integration [83,171,172]. These observations indicate that the safety profile of S/MAR-based vectors is context-specific. Other hybrid systems rely on recombination machinery, often co-delivered using a second vector, to release the nonviral episomes from the viral vector backbone [165,170]. The high recombinase concentrations needed for efficient release and the high vector doses required within a two-vector system bear the risk of recombinase-dependent genotoxicity and vector-dependent immunogenicity (reviewed in [173,174]). Thus, detailed analyses for each particular viral hybrid system are inevitable since the transferability of safety profiles of their nonhybrid counterparts is limited.

Presumably, developing new production systems for viral vectors might foster the engineering of novel vectors combining ideal features from both worlds. As discussed in this review, recent developments in nonviral vector designs rendered S/MAR-based episomes and SB transposases able for their use in *ex vivo* gene therapy applications. Both vector systems were successfully used for the genetic modification of patient-derived T-cells, and SB is currently involved in respective clinical trials [75,163]. However, for a broader field of applications, hybrid viral vectors may represent a reemerging perspective regarding efficiency and safety.

Although currently approved viral vectors are highly efficient in terms of delivery per se, the lack of specific targeting strategies requires the administration of high vector doses. Moreover, the fact that only a fraction of applied vector particles reach the intended tissue or organ demands the use of strong promoters facilitating high transgene expression level that often exceeds the expression level of the endogenous gene. Especially the administration of high vector doses led in the past and recently to severe immunologic adverse effects and fatalities [8,175,176]. Thus, for future gene therapy approaches, it is imperative to consider targeting strategies to reduce vector doses and the need for high-level transgene expression. Within the last decade, an enduring toolbox for modifying viral capsids has been developed. In addition to genetic approaches, the chemical and genetic-chemical modification of viral capsids allows different de- and re-targeting strategies, enabling not only the targeted delivery of viral vectors but also reduced immunological side and off-target effects [177] (and reviewed in [5]). Thus, complementing the recent progress in the design of nonviral vectors with the versatile toolbox of viral delivery strategies is a valuable contribution to future developments in vector design.

## Figures and Tables

**Table 1:** Advantages and disadvantages of nonviral vector systems. The major limitation of each vector system is marked in bold. The term delivery efficiency, as used here, refers to the ratio of the copy number of genetic material that becomes established/integrated into target cells to the copy number of genetic material used for initial transfection/transduction/injection.

VECTOR SYSTEM	ADVANTAGES	DISADVANTAGES
**EBV-based replicons**	- stable transgene expression - high cloning capacity (>100 kb) - episomal maintenance - efficient modification of target cells - easy engineering, low-cost production	- contains viral elements - low delivery efficiencies (in vitro and in vivo) - limited targeting options - EBNA-1-related toxicity
**Sleeping Beauty**	- stable transgene expression - comprehensively studied safety profile - efficient modification of target cells	- risk of insertional mutagenesis - requires co-delivery of transposases - low delivery efficiencies (in vitro and in vivo)
**S/MAR-based replicons**	- lack of viral elements - episomal maintenance - easy engineering, low-cost production - low immunogenicity	- low establishment efficiencies - limited cloning capacity - low delivery efficiencies (in vitro and in vivo) - limited targeting options
**Artificial Chromosomes**	- unlimited cloning capacity - copy number control - nonintegrating - nonessential, additional chromosome	- complex construction - low delivery efficiencies (in vitro and in vivo) - construct-dependent inefficiencies in segregation to daughter cells possible - potential risks imposed by homologous recombination need to be analysed in depth

## Data Availability

Not applicable.
